# Overall obesity, abdominal adiposity, diabetes and cigarette smoking in relation to the risk of pancreatic cancer in two Swedish population-based cohorts

**DOI:** 10.1038/sj.bjc.6602868

**Published:** 2005-11-15

**Authors:** S C Larsson, J Permert, N Håkansson, I Näslund, L Bergkvist, A Wolk

**Affiliations:** 1 Division of Nutritional Epidemiology, The National Institute of Environmental Medicine, Karolinska Institutet, Box 210, SE-17177 Stockholm, Sweden; 2 Department for Clinical Sciences, Intervention and Technology, Karolinska Institutet, Karolinska University Hospital, Huddinge, SE-14186 Stockholm, Sweden; 3 Department of Surgery, Örebro University Hospital, SE-17185 Örebro, Sweden; 4 Department of Surgery and Centre for Clinical Research, Central Hospital, SE-72189 Västerås, Sweden

**Keywords:** anthropometry, body mass index, cohort studies, diabetes mellitus, pancreatic neoplasms, obesity, prospective studies, smoking, smoking cessation

## Abstract

We examined the associations of body mass index (BMI), waist circumference, a history of diabetes, and cigarette smoking with risk of pancreatic cancer among 37 147 women and 45 906 men followed up during 560 666 person-years in the Swedish Mammography Cohort and the Cohort of Swedish Men; 136 incident cases of pancreatic cancer were diagnosed. The multivariate rate ratio (RR) of pancreatic cancer for obese women and men (BMI ⩾30 kg/m^2^) was 1.81 (95% CI: 1.04–3.15) compared to those with a BMI of 20–25 kg/m^2^. For a difference of 20 cm (about two standard deviations) in waist circumference, the multivariate RRs were 1.32 (95% CI: 0.73–2.37) among women and 1.74 (95% CI: 1.00–3.01) among men. Pancreatic cancer risk was associated with history of diabetes (multivariate RR: 1.88; 95% CI: 1.09–3.26) and cigarette smoking (multivariate RR for current compared with never smokers: 3.06; 95% CI: 1.99–4.72). Current smokers of ⩾40 pack-years had a five-fold elevated risk compared with never smokers. Risk among past smokers approached the RR for never smokers within 5–10 years following smoking cessation. Findings from this prospective study support positive relationships of overall obesity, abdominal adiposity, diabetes and smoking with risk of pancreatic cancer.

Cancer of the pancreas, the sixth leading cause of cancer death in the European Union (Boyle and Ferlay, 2005), is a rapidly fatal malignancy. Less than 5% of patients survive 5 years after diagnosis (Sant *et al*, 2003). Primary prevention of pancreatic cancer is therefore of particular importance. Unfortunately, the etiology of pancreatic cancer remains largely elusive. Cigarette smoking is the only generally accepted modifiable risk factor, but explains only 25–29% of pancreatic cancer incidence (Silverman *et al*, 1994; Fuchs *et al*, 1996).

Evidence from *in vitro*, animal and human studies indicates that insulin, insulin resistance and abnormal glucose metabolism may play a role in pancreatic cancer etiology (Fisher *et al*, 1996; Gapstur *et al*, 2000; Schneider *et al*, 2001; Jee *et al*, 2005). Moreover, epidemiological studies have suggested a relationship between diabetes mellitus and increased risk of pancreatic cancer (Huxley *et al*, 2005). Obesity, and specifically abdominal adiposity, has been linked to metabolic abnormalities, including insulin resistance, hyperinsulinemia, glucose intolerance and to the development of diabetes mellitus (Kahn and Flier, 2000; IARC, 2002). Thus, obesity may be a risk factor for pancreatic cancer. However, epidemiological studies of body mass index (BMI), as a measure of overall obesity, in relation to risk of pancreatic cancer have yielded inconsistent results with a positive association observed in some, but not all studies (Berrington de Gonzalez *et al*, 2003). To our knowledge, no study has investigated the association between waist circumference, as an estimate of abdominal adiposity, and risk of pancreatic cancer.

In two prospective population-based cohorts of Swedish women and men, we examined the relationships between overall obesity (reflected by BMI) and abdominal adiposity (waist circumference) and the risk of pancreatic cancer. In addition, we report findings for diabetes, cigarette smoking and smoking cessation on pancreatic cancer risk.

## MATERIALS AND METHODS

### Study population

Two population-based prospective cohort studies provided data for the present analyses: the Swedish Mammography Cohort (SMC) and the Cohort of Swedish Men (COSM). The SMC was established between 1987 and 1990, when all women born between 1914 and 1948 and living in central Sweden (Västmanland and Uppsala counties) received a mailed questionnaire that elicited information on diet, weight, height and education; 66 651 women (74% of the source population) returned a completed questionnaire. In the autumn of 1997, an expanded questionnaire that included data on various lifestyle factors and medical history was mailed to women who were still alive and residing in the study area; 39 227 women (70%) completed the questionnaire. In the autumn of 1997, 48 850 men born between 1918 and 1952 and residing in central Sweden (Västmanland and Örebro counties) were enrolled in the COSM by return of a mailed questionnaire that was identical to the 1997 SMC questionnaire (except for some sex-specific questions).

Eligible participants for the present study were women and men who completed the 1997 questionnaire (information on cigarette smoking and diabetes was first obtained in 1997). After excluding participants with erroneous or missing national registration number and those with a cancer diagnosis (except nonmelanoma skin cancer) before baseline, a total of 37 147 women and 45 906 men were eligible for follow-up. The study was approved by the Regional Ethical Review Board in Stockholm.

### Assessment of exposures

In 1997, participants completed a self-administered questionnaire that included information on demographic characteristics, weight, height, waist circumference, cigarette smoking status and history, alcohol consumption, dietary intake, physical activity and history of diabetes mellitus; complementary information on diabetes for subjects who were hospitalized was obtained from the Swedish Inpatient Register. Pack-years were estimated from baseline smoking history by multiplying the number of years of smoking by the average number of packs of cigarettes smoked. We estimated BMI from self-reported weight and height (kg/height in m^2^) as a measure of overall obesity. High validity has been observed for self-reported height (*r*=0.9) and weight (*r*=0.9) compared with actual measurement among Swedish women and men (Kuskowska-Wolk *et al*, 1989). We used self-reported waist circumference as an estimate of abdominal adiposity. We do not have validity data for self-report of waist from the Swedish population, but results from an UK population have indicated high validity (*r*=0.8) (Spencer *et al*, 2004).

### Ascertainment of cases and follow-up

Incident cases of pancreatic cancer were identified by computerised record linkage of the study population to the National Swedish Cancer Register and the Regional Cancer Register covering the study area, both of which have been estimated to be nearly 100% complete (Mattsson and Wallgren, 1984). Pancreatic cancer cases were defined as primary malignant neoplasm of the exocrine pancreas [*International Classification of Diseases, Ninth Revision* (ICD-9) code 157]. We excluded islet-cell carcinomas (ICD-9 code 157.4) because the etiology of these tumors may be different from that of the exocrine pancreas. Information on dates of death and dates of moving out of the study area was obtained from the Swedish Death and Population registries.

### Statistical analysis

Person-years of follow-up for each participant were computed as the time from baseline to the date of diagnosis of pancreatic cancer, death from any cause, emigration, or until December 31, 2004. Participants were categorized into four groups with BMI (kg/m^2^) corresponding to <20.0, 20.0–24.9 (reference group), 25.0–29.9 and ⩾30.0. Participants with a BMI below 15 or above 60 kg/m^2^ were excluded in the BMI analyses. For waist circumference, we used quartiles based on the distribution among women and men separately.

Cox proportional hazards models (Cox and Oakes, 1984) were used to estimate rate ratios (RRs) with 95% confidence intervals (CIs). Age (in months) and sex were controlled for as stratification variables in the Cox model. In multivariate models, we included education (less than high school, high-school graduate, or more than high school), BMI (<20.0, 20.0–25.0, 25.0–29.9, or ⩾30 kg/m^2^), physical activity (four categories), history of diabetes (yes or no), cigarette smoking (never, past <20 pack-years, past ⩾20 pack-years, current <20 pack-years, current 20–39 pack-years, or current ⩾40 pack-years) and alcohol consumption (quartiles). Multivariate analyses of waist circumference were further controlled for height (quartiles). Trends tests for BMI and waist circumference were performed by scoring the categories and entering the score as a continuous term in the regression model. Statistical interaction between BMI and cigarette smoking in relation to pancreatic cancer risk was tested using the likelihood ratio test. We used the SAS statistical package (version 9.1; SAS Institute, Inc., Cary, NC, USA) for all analyses. All statistical tests are two-sided.

## RESULTS

A total of 136 incident cases (61 females and 75 males) of pancreatic cancer were diagnosed during 560 666 person-years of follow-up, yielding a crude incidence rate of 24.3 per 100 000 person-years. The incidence rate was similar among women (24.1 per 100 000 person-years) and men (24.4 per 100 000 person-years). The mean (±s.d.) age of the participants at baseline in 1997 was 62 (9.3) years for women and 60 (9.7) years for men. The mean (±s.d.) age of the participants at time of diagnosis of pancreatic cancer was 72 (8.5) years for female cases and 72 (8.0) years for male cases. Table 1 presents the baseline characteristics by BMI. The mean (±s.d.) baseline BMI was 25.0 (3.9) kg/m^2^ among women and 25.8 (3.4) kg/m^2^ among men. In all, 34% percent of the women and 46% of the men were overweight (BMI 25–29.9 kg/m^2^); 11% percent of the women and 10% of the men were obese (BMI ⩾30 kg/m^2^). Compared with women and men with a BMI of 20–25 kg/m^2^, obese women and men were less likely to have a post-secondary education and to be current smokers, but were more likely to have diabetes; they also exercised less and had lower alcohol consumption. BMI was strongly correlated with waist circumference (women, *r*=0.76; men, *r*=0.75). The proportions of never, past and current smokers, respectively, at baseline were 54, 27 and 19% among women and 36, 46 and 18% among men.

We observed a positive association between BMI and risk of pancreatic cancer (Table 2). After controlling for age and other potential risk factors for pancreatic cancer, women and men with a BMI of 30 kg/m^2^ or higher had an 81% increased risk compared with those with a BMI of 20–25 kg/m^2^. When analysed as a continuous variable, an increment of 1 BMI unit (kg/m^2^) was related to a 5% increased risk of pancreatic cancer among women and men combined (RR: 1.05; 95% CI: 1.00–1.10), a 4% increased risk among women (RR: 1.04; 95% CI: 0.97–11), and a 6% increased risk among men (RR: 1.06; 95% CI: 0.99–1.14). The findings for BMI did not change appreciably after excluding cases that occurred within the first year of follow-up. Waist circumference was also positively associated with risk of pancreatic cancer, and similar to the observed association for BMI; the risk was greater among men (Table 2). For a difference of 20 cm (approximately two standard deviations) in waist circumference, the multivariate RRs were 1.32 (95% CI: 0.73–2.37) among women and 1.74 (95% CI: 1.00–3.01) among men.

A history of diabetes was associated with a statistically significant 1.9-fold elevated risk of pancreatic cancer (Table 2). This association remained after early follow-up cancers (i.e., those diagnosed within the first year) were excluded (RR: 1.97; 95% CI: 1.10–3.53). The association with diabetes seemed to be confined to men, but the number of diabetic women with pancreatic cancer was small (*n*=5).

Women and men who were current smokers at baseline had a significantly higher risk of pancreatic cancer compared with never smokers (Table 3). Past smoking was not related to a significantly increased risk, although women who had smoked in the past had a nonsignificant elevation in risk. Risk of pancreatic cancer increased with increasing number of pack-years of smoking. Current smokers of 40 or more pack-years had a fivefold elevated risk of pancreatic cancer compared to never smokers. An increment of 10 pack-years of smoking was associated with a multivariate RR of 1.36 (95% CI: 1.15–1.61; *P* value for trend=0.0003).

**Figure 1:**
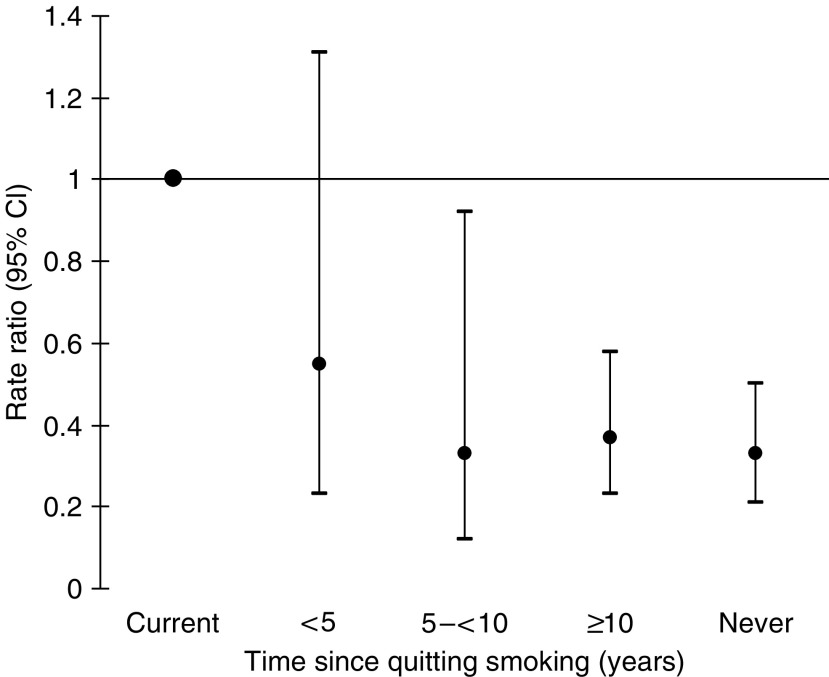
Multivariate rate ratios of pancreatic cancer in relation to time since quitting of smoking among women and men. Multivariate models were stratified by age in months at baseline and sex and were simultaneously adjusted for education (less than high school, high school graduate, or more than high school), body mass index (<20.0, 20.0–24.9, 25.0–29.9, or ⩾30 kg/m^2^), physical activity (four categories), history of diabetes (yes or no), and alcohol consumption (quartiles).

We also assessed the association between smoking cessation and risk of pancreatic cancer (Figure 1). Compared with current smokers, the RR among past smokers diminished steeply and approached the RR for never smokers within 5–10 years following smoking cessation.

**Figure 2:**
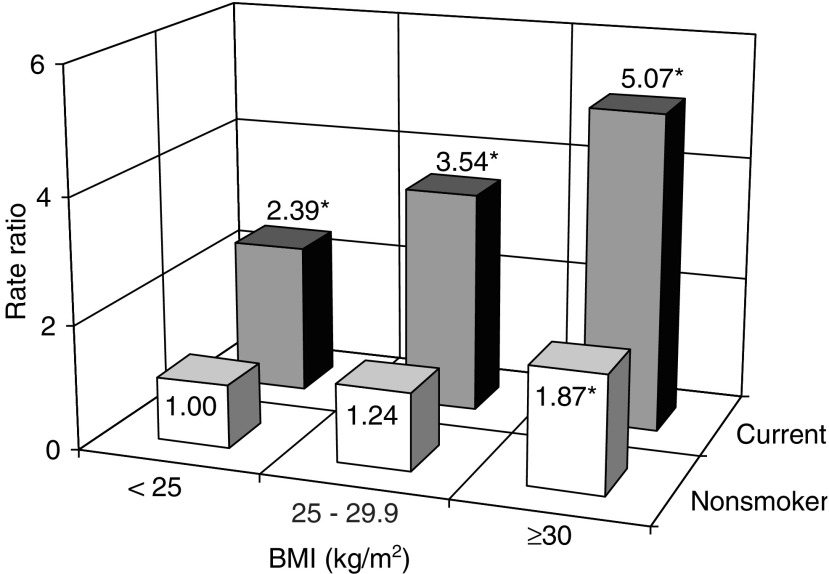
Multivariate rate ratios of pancreatic cancer according to body mass index (BMI) and cigarette smoking status among women and men. Multivariate models were stratified by age in months at baseline and sex and were simultaneously adjusted for education (less than high school, high school graduate, or more than high school), physical activity (four categories), history of diabetes (yes or no), and alcohol consumption (quartiles). Nonsmoker include never and past smokers. ^*^Statistically significant.

Figure 2 shows the RRs of pancreatic cancer according to cross-classification of BMI and smoking status. High BMI and cigarette smoking were independently associated with increased risk of pancreatic cancer and there was no apparent effect modification between these variables (*P* for interaction=0.74). Compared with nonsmokers (never and past) with a BMI of less than 25 kg/m^2^, current smokers with a BMI of 30 kg/m^2^ or more, had a multivariate RR of 5.07 (CI: 1.93–13.30).

## DISCUSSION

In this prospective analysis, we observed approximately a doubling of the risk of pancreatic cancer for obese women and men (BMI ⩾30 kg/m^2^) compared with those with a normal weight (BMI 20–25 kg/m^2^). We also confirmed associations between history of diabetes and cigarette smoking and increased risk of pancreatic cancer. In addition, we found that smokers who had quit smoking for 5–10 years had a risk of developing pancreatic cancer similar to that of never smokers.

To our knowledge, this was the first study to evaluate the relationship between waist circumference (reflecting abdominal adiposity) and risk of pancreatic cancer. We found that waist circumference was positively associated with pancreatic cancer risk, particularly among men. A previous cohort study found that women and men who reported ‘central’ weight gain had increased relative risk of pancreatic cancer compared with women and men who reported peripheral weight gain; the association was stronger among men (Patel *et al*, 2005).

Previous studies of BMI and pancreatic cancer risk have produced inconsistent results. A recent meta-analysis of 14 studies on obesity and risk of pancreatic cancer estimated a 19% increase in risk among obese individuals compared with those with a normal weight (RR: 1.19; 95% CI: 1.10–1.29 for BMI 30 *vs* 22 kg/m^2^) (Berrington de Gonzalez *et al*, 2003). The summary risk estimate was higher when the authors excluded studies that did not control for smoking; the estimate was also higher for cohort than for case–control studies (Berrington de Gonzalez *et al*, 2003). Since that meta-analysis, two case–control studies based on direct patient interviews (Pan *et al*, 2004; Fryzek *et al*, 2005) and two cohort studies (Samanic *et al*, 2004; Patel *et al*, 2005) have reported a statistically significant positive relation between BMI and pancreatic cancer risk in both women and men or only in men. Another cohort of elderly women observed no increase in pancreatic cancer risk with greater BMI (Sinner *et al*, 2005).

In the present study, we observed a 1.9-fold higher risk of pancreatic cancer among women and men with diabetes. A meta-analysis of 17 case–control and 19 cohort or nested case–control studies (Huxley *et al*, 2005) showed a 1.8-fold higher risk of pancreatic cancer associated with a history of diabetes; the magnitude of the positive association was greater among individuals whose diabetes had been recently diagnosed.

A metabolic consequence of obesity, particularly the accumulation of intra-abdominal fat, is the development of insulin resistance, which leads to an increase in the secretion of insulin from the pancreas (IARC, 2002). A role of hyperinsulinemia in pancreatic pathogenesis might be through an increase in local blood flow and cell division in the pancreas (Henderson *et al*, 1981). The exocrine pancreatic cells are exposed to very high insulin concentrations through a portal circulation system from the insulin-producing pancreatic islets (Williams and Goldfine, 1985). High concentrations of insulin are able to activate the insulin-like growth factor I (IGF-I) receptor, and activation of this receptor leads to growth-promoting effects (Le Roith, 1997). Furthermore, excess insulin, through downregulation of the insulin-like growth factor binding protein-1, could result in an increase in the exposure to free IGF-I (Giovannucci, 2003). Both insulin and IGF-I have been shown to promote growth in pancreatic cell lines (Ohmura *et al*, 1990; Takeda and Escribano, 1991; Bergmann *et al*, 1995; Fisher *et al*, 1996).

This study has several strengths. The prospective design precluded recall bias and the need to use next-of-kin respondents. In addition, we had virtually complete follow-up as incident cases of pancreatic cancer were ascertained by record linkage to the Swedish Cancer Registry. Differential follow-up is therefore unlikely to have affected our results. We were also able to control for potential confounding by most known or possible risk factors for pancreatic cancer. Because data regarding exposures were collected before the diagnosis of pancreatic cancer, any misclassification would be nondifferential and would most likely have weakened rather than exaggerated any true associations. A limitation of this study is the self-reported information on anthropometric measures, diabetes and cigarette smoking. Overweight and obese individuals tend to underestimate their body size (weight, BMI, waist) to a greater extent than those who are lean, and shorter individuals tend to overestimate their height (Kuskowska-Wolk *et al*, 1989; Spencer *et al*, 2004). These biases are likely to attenuate risk estimates of the relations between BMI and waist circumference and risk of pancreatic cancer; thus the associations may be even stronger in the absence of measurement error. Another limitation of our study is the lack of information on timing of diagnosis of diabetes.

In summary, results from this population-based prospective study suggest that obesity may increase the risk for pancreatic cancer. Findings from this study also provide further support for associations of history of diabetes and cigarette smoking with pancreatic cancer risk.

## Figures and Tables

**Table 1:** Age-standardised characteristics among women and men according to categories of body mass index at baseline

	**Body mass index (kg/m^2^)**			
**Characteristics**	**<20.0**	**20.0–24.9**	**25.0–29.9**	**⩾30.0**
*Women*				
Age in years, mean	62.6	61.0	62.6	62.1
Postsecondary education (%)	24.8	21.4	15.8	12.8
Current smokers (%)	30.8	20.7	17.1	14.8
Pack-years ever smoked (past or current), mean	16.2	14.4	15.3	16.5
Exercise (hours/week)	2.6	2.5	2.3	2.0
History of diabetes (%)	2.3	2.7	4.8	9.5
Waist circumference, mean (cm)	72.2	79.1	88.2	99.3
Alcohol consumption, mean (g/day)	4.1	4.2	3.6	2.7
				
*Men*				
Age in years, mean	62.2	60.1	60.1	59.7
Postsecondary education (%)	23.4	19.9	14.5	11.1
Current smokers (%)	32.5	19.7	16.5	16.8
Pack-years ever smoked (past or current), mean	24.4	19.1	19.8	22.4
Exercise (hours/week)	2.4	2.7	2.5	2.2
History of diabetes (%)	3.8	4.2	6.2	12.8
Waist circumference, mean (cm)	82.5	90.2	98.9	110.9
Alcohol consumption, mean (g/day)	9.9	10.5	10.3	9.1

**Table 2:** Rate ratios (95% confidence intervals) of pancreatic according to body mass index, waist circumference, and diabetes

	**Women**			**Men**			**Woman and men**
	**Cases^a^**	**Age-adjusted RR**	**Multivariate RR^b^**	**Cases^a^**	**Age-adjusted RR**	**Multivariate RR^b^**	**Multivariate RR^b^,^c^**
*Body mass index (kg/m^2^)*							
<20.0	3	0.94 (0.28–3.16)	0.76 (0.22–2.59)	2	1.46 (0.34–6.20)	1.54 (0.35–6.66)	0.96 (0.38–2.46)
20.0–24.9	24	1.00 (reference)	1.00 (reference)	26	1.00 (reference)	1.00 (reference)	1.00 (reference)
25.0–29.9	25	1.44 (0.82–2.54)	1.57 (0.87–2.81)	29	1.02 (0.60–1.73)	1.06 (0.62–1.82)	1.25 (0.84–1.86)
⩾30.0	7	1.32 (0.56–3.08)	1.48 (0.60–3.62)	12	1.99 (1.00–3.98)	2.08 (1.02–4.25)	1.81 (1.04–3.15)
*P* for trend		0.27	0.12		0.21	0.17	0.04
							
*Waist circumference* d							
Quartile 1	8	1.00 (reference)	1.00 (reference)	8	1.00 (reference)	1.00 (reference)	1.00 (reference)
Quartile 2	8	0.87 (0.32–2.33)	0.94 (0.34–2.58)	12	1.29 (0.52–3.16)	1.36 (0.55–3.36)	1.15 (0.59–2.25)
Quartile 3	18	1.45 (0.62–3.38)	1.77 (0.74–4.22)	16	1.36 (0.58–3.20)	1.48 (0.62–3.51)	1.59 (0.87–2.93)
Quartile 4	15	1.18 (0.49–2.84)	1.46 (0.58–3.66)	21	1.94 (0.85–4.40)	2.00 (0.85–4.66)	1.72 (0.93–3.20)
*P* for trend		0.46	0.23		0.10	0.10	0.05
							
*History of diabetes*							
No	57	1.00 (reference)	1.00 (reference)	64	1.00 (reference)	1.00 (reference)	1.00 (reference)
Yes	5	1.42 (0.56–3.60)	1.31 (0.49–3.47)	11	2.13 (1.11–4.09)	2.32 (1.19–4.53)	1.88 (1.09–3.26)

a The number of cases may not sum up to the total number of cases owing to missing data on BMI or waist circumference.

b Multivariate models were stratified by age in months at baseline and were simultaneously adjusted for education (less than high school, high school graduate, and more than high school), physical activity (four categories), cigarette smoking (never, past <20 pack-years, past ⩾20 pack-years, current <20 pack-years, current 20–39 pack-years, or current ⩾40 pack-years), and alcohol consumption (quartiles). Analyses of body mass index were further adjusted for diabetes; analyses of waist circumference were further adjusted for diabetes and height (quartiles); analyses of diabetes were further adjusted for body mass index.

c Also adjusted for sex.

d Cut points for quartiles of waist circumference (cm) for women were <76, 76–81, 82–89, and ⩾90; for men <90, 90–94, 95–101, and ⩾102.

**Table 3:** Rate ratios (95% confidence intervals) of pancreatic cancer according to cigarette smoking status and total number of pack-years of smoking

	**Women**			**Men**			**Woman and men**
	**Cases**	**Age-adjusted RR**	**Multivariate RR^a^**	**Cases**	**Age-adjusted RR**	**Multivariate RR^a^**	**Multivariate RR^a^,^b^**
*Smoking status*							
Never smoked	26	1.00 (reference)	1.00 (reference)	22	1.00 (reference)	1.00 (reference)	1.00 (reference)
Past smoker	13	1.48 (0.75–2.93)	1.39 (0.69–2.78)	32	1.10 (0.64–1.89)	1.00 (0.57–1.74)	1.16 (0.75–1.80)
Current smoker	22	3.44 (1.91–6.19)	3.81 (2.08–7.00)	21	2.52 (1.38–4.63)	2.47 (1.33–4.55)	3.06 (1.99–4.72)
							
*Pack-years,*^c^ *past smokers*							
Never smoked	26	1.00 (reference)	1.00 (reference)	22	1.00 (reference)	1.00 (reference)	1.00 (reference)
<20	7	0.95 (0.41–2.22)	0.90 (0.38–2.12)	18	0.90 (0.48–1.69)	0.84 (0.44–1.58)	0.92 (0.56–1.53)
⩾20	6	4.36 (1.75–10.89)	4.15 (1.63–10.51)	14	1.52 (0.77–2.98)	1.33 (0.67–2.66)	1.91 (1.08–3.35)
							
*Pack-years,*^c^ *current smokers*							
Never smoked	26	1.00 (reference)	1.00 (reference)	22	1.00 (reference)	1.00 (reference)	1.00 (reference)
<20	8	2.38 (1.06–5.34)	2.65 (1.16–6.05)	7	2.33 (0.98–5.53)	2.30 (0.97–5.50)	2.46 (1.35–4.49)
20–39	11	4.21 (2.02–8.78)	4.81 (2.27–10.18)	5	1.54 (0.58–4.13)	1.53 (0.57–4.11)	2.91 (1.62–5.25)
⩾40	3	7.71 (2.24–26.59)	6.90 (1.91–24.93)	9	4.23 (1.92–9.33)	4.05 (1.81–9.06)	5.13 (2.60–10.11)

a Multivariate models were stratified by age in months at baseline and were simultaneously adjusted for education (less than high school, high school graduate, and more than high school), body mass index (<20.0, 20.0–24.9, 25.0–29.9, or ⩾30 kg/m^2^), physical activity (four categories), history of diabetes (yes, no), and alcohol consumption (quartiles).

b Also adjusted for sex.

c Number of packs of cigarettes smoked per day (one pack contains 20 cigarettes) multiplied by the number of years of smoking.
